# Direct oral anticoagulants versus low-molecular-weight heparin for thromboprophylaxis in cancer-related surgeries: A meta-analysis of efficacy and safety outcomes

**DOI:** 10.1016/j.ahjo.2025.100607

**Published:** 2025-09-11

**Authors:** Asma Mousavi, Shayan Shojaei, Parham Dastjerdi, Soheil Rahmati, Kasra Izadpanahi, Homayoun Pishraft-sabet, Elmira Jafari Afshar, Keyvan Salehi, Mahshad Sabri, Mahsa Noohi Arbatan, Parisa Fallahtafti, Aronow Wilbert, Andrew P. Ambrosy, Mushabbar A. Syed, Mina Iskander, Kaveh Hosseini

**Affiliations:** a Tehran Heart Center, Cardiovascular Diseases Research Institute, Tehran University of Medical Sciences, Tehran, Iran; b School of Medicine, Tehran University of Medical Sciences, Tehran, Iran; c Students' Scientific Research Center, Tehran University of Medical Sciences, Tehran, Iran; d Department of Cardiovascular Diseases, Faculty of Medicine, Mashhad University of Medical Sciences, Mashhad, Iran; e Cardiovascular Research Center, Alborz University of Medical Sciences, Alborz, Iran; f School of Medicine, Shahid Beheshti University of Medical Sciences, Tehran, Iran; g Rajaei Cardiovascular Medical and Research Center, Iran University of Medical Sciences, Tehran, Iran; h Westchester Medical Center, Cardiology Division, New York Medical College, Valhalla, New York, United States; i Kaiser Permanente, San Francisco Medical Centre and Medical Offices, San Francisco, United States; j Loyola University Stritch School of Medicine, Chicago, United States; k Heart and Vascular Center, Center of Advanced Care, Froedtert Hospital, WI, United States

**Keywords:** DOACs, LMWH, Cancer-related surgeries, Thromboprophylaxis, meta-analysis

## Abstract

**Background:**

Post-operative venous thromboembolism (VTE) remains a concern following cancer-related surgeries. This systematic review and meta-analysis aimed to evaluate the safety and efficacy of direct oral anticoagulants (DOACs) compared to low molecular weight heparin (LMWH) for thromboprophylaxis after cancer-related surgeries.

**Methods:**

We systematically searched databases for studies comparing DOACs to LMWH for post-operative thromboprophylaxis in patients undergoing cancer-related surgeries. Primary outcomes were VTE incidence and bleeding events. Secondary outcomes included all-cause mortality and hospitalization rates. Subgroup analyses examined DOAC type, cancer type, and follow-up duration. A random-effects model calculated pooled risk ratios (RRs) with 95 % confidence intervals (CIs).

**Results:**

Analysis included 16 studies with 6400 participants in the DOAC group (mean age 62.05 years, 28.15 % male) and 5801 participants in the LMWH group (mean age 60.78 years, 34.65 % male). DOACs were non-inferior to LMWH for VTE prevention (RR = 0.81, 95 % CI 0.56 to 1.16) with no significant difference in bleeding rates (RR = 0.70, 95 % CI 0.70 to 1.18). Mortality and hospitalization rates were similar between groups. Subgroup analyses suggested possible VTE reduction with DOACs in urological cancer surgeries (RR = 0.52, 95 % CI 0.44 to 0.61) and lower bleeding trends with Apixaban (RR = 0.64, 95 % CI 0.44 to 0.94).

**Conclusions:**

DOACs appear non-inferior to LMWH for post-operative thromboprophylaxis in patients undergoing cancer-related surgeries, with comparable safety. The superior VTE prevention in urological cancer surgeries and Apixaban's favorable safety profile warrant further investigation. Moreover, additional research is necessary to clarify the roles of specific DOACs and optimal prophylaxis strategies across various cancer types and surgical procedures.

## Highlights

• DOACs were found to be non-inferior to LMWH in preventing venous thromboembolism (VTE) post-operatively in cancer patients.
• The incidence of bleeding, mortality, and hospitalization was generally similar between DOACs and LMWH.
• DOACs have shown comparable efficacy to LMWH in preventing VTE across various cancer types.
• In the subgroup analysis, Apixaban significantly reduced the risk of major bleeding compared to LMWH.

## Graphical abstract

**Figure:**
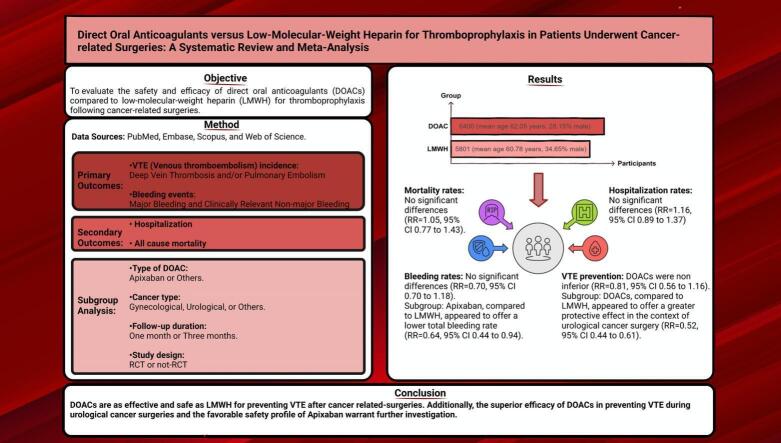

## 1 Introduction

Venous thromboembolism (VTE), comprised of deep vein thrombosis (DVT) and pulmonary embolism (PE), is the second cause of death in patients with cancer overall [1]. Studies have demonstrated that patients with cancer have a significantly higher risk of VTE [2] and are more likely to exhibit recurrence and bleeding during anticoagulant therapy than those without malignancy [3]. Moreover, as a common method for cancer treatment, surgery is associated with an increased risk of postoperative VTE events for cancers [4,5]. Given the procoagulant nature of malignancies and surgical interventions, anticoagulation recommendations are crucial in managing these patients [6,7].

Clinical guidelines suggest that all patients with malignant tumors undergoing surgical intervention should receive pharmacologic thromboprophylaxis with either unfractionated heparin (UFH) or low-molecular-weight heparins (LMWHs) unless there are contraindications [[8], [9], [10], [11]]. These interventions should be continued for a minimum of 7–10 days, while extended prophylaxis up to 4 weeks postoperatively is recommended in patients with cancer who have gone under major open or laparoscopic abdominal or pelvic surgeries. Besides LMWH, UFH, and other parenterally injected anticoagulants, there are also oral antithrombotic agents, for instance, direct oral anticoagulants (DOACs) [12]. Compared to LMWHs, there are several advantages to DOAC therapies, such as oral administration [13], fixed doses, and lower cost [14], while there are some concerns about gastrointestinal or major bleeding [15].

Currently, using DOACs as a safe and effective option is strongly recommended by clinical guidelines for cancer-associated thrombosis (CAT), but their safety and efficacy in surgical oncology are still unclear [[8], [9], [10], [11]]. Recent randomized controlled trials (RCTs) comparing LMWHs versus DOACs have shown similar outcomes in patients who have undergone cancer-related surgery, with higher satisfaction in DOAC groups [16,17]. Although recently published systematic reviews and meta-analyses demonstrated no significant difference between these treatments in terms of outcomes [18,19], the current clinical practice guidelines highlight insufficient evidence to support the application of DOACs as an alternative to LMWHs for the thromboprophylaxis of postoperative VTE in patients with cancer [8,9,11]. Updated ASCO guideline in 2023 proposes two DOACs (Apixaban and Rivaroxaban) could be used as an alternative after an initial period of LMWH or UFH for extended thromboprophylaxis after cancer-related surgery but with a weak strength of recommendation because the included evidence had limitations in sample size and differed in patient population and timing of intervention [20]. As the clinical practice in this setting has remained uncertain, we aimed to conduct a systematic review and meta-analysis of published RCTs and observational studies to compare the safety and efficacy of postoperative LMWHs and DOACs as thromboprophylaxis in patients who underwent cancer-related surgery to help better evidence-based decision-making and treatment.

## 2 Methods

### 2.1 Protocol

This systematic review and meta-analysis was conducted in accordance with the Preferred Reporting Items for Systematic Reviews and Meta-Analyses (PRISMA) guidelines and the Cochrane guidelines [21]. The study protocol was registered in the International Prospective Register for Systematic Reviews (PROSPERO) (registration code: CRD42025634920).

### 2.2 Search strategy

A systematic literature search was performed using EMBASE, PubMed, Scopus, and Web of Science without time limitation. An online search for completed and ongoing research, as well as references of related reviews and included trials, was also conducted. The complete search strategy is available in Supplemental Table 1.

Four investigators (H.P., S.R., K.I., P.D.) independently selected the title, abstract, and full text of the studies. Two different authors (A.M. and S.S.) reviewed the discrepancies and resolved them by consensus. The screening process was conducted using Rayyan [22].

### 2.3 Inclusion and exclusion criteria

Articles meeting the following criteria were included: (1) randomized controlled trials (RCTs) and observational studies; [23] adult patients (18 years old or older) who underwent cancer-related surgery; and (3) directly compared DOAC (Dabigatran, Rivaroxaban, Apixaban, Betrixaban, or Edoxaban) to LMWH (Dalteparin, Enoxaparin, Tinzaparin, or Nadroparin) as thromboprophylaxis after surgery. The meta-analysis excluded case reports, review articles, descriptive articles, animal trials, surgeries unrelated to cancer, non-comparative observational studies, studies that did not compare DOACs with LMWH, conference abstracts, and those where anticoagulation was initiated for treatment rather than prophylaxis. We also excluded studies that involved non-surgical cancer patients.

### 2.4 Outcome measures

Post-operative VTE, defined as asymptomatic or symptomatic DVT and/or PE, was considered the primary efficacy outcome. Major bleeding and clinically relevant non-major bleeding (CRNMB), defined according to the International Society on Thrombosis and Hemostasis [24,25] were identified as the primary safety outcomes. Secondary outcomes encompass all-cause mortality and hospitalization rates.

### 2.5 Risk of bias assessment

The quality of RCTs was assessed using the Cochrane Risk of Bias 2 (RoB2) tool [26], and observational studies were assessed using the Newcastle–Ottawa Scale (NOS) [27]. This assessment was performed by two investigators independently (M.S. and A.M.) and disparities were resolved by seeking the advice of a third author (A.M.). The RoB2 tool evaluates RCTs across five domains: randomization process, deviation from intended interventions, missing outcome data, measurement of outcomes, and selection of reported results. The NOS tool assesses original studies across three main domains: selection, comparability, and outcome.

### 2.6 Data extraction

Two authors (P.D. and E.J.) independently assessed the included studies and extracted relevant data, including study characteristics (first author, publication year, country, study design, inclusion and exclusion criteria, cancer type, surgery type, and follow-up period), baseline characteristics of participants (medication name and dosage, indication for anticoagulant initiation, days after surgery that medication initiated, number of participants in case and control groups, mean age, gender percentage, a previous bleeding event leading to hospitalization or severe condition, previous VTE, and previous ambulation issues, cancer stage, cancer metastasis, chemotherapy, and radiotherapy), and outcomes (VTE, PE, DVT, bleeding, mortality, and hospital readmission). The extracted data were then rechecked by A.M.

The case group in our study consisted of DOAC, and the control group consisted of LMWH. Subgroup analyses were conducted based on cancer various types (gynecological, urological, and others), follow-up period (1 month or 3 months), anticoagulant type (pixaban or other DOACs), and study design (RCT or not RCT).

### 2.7 Statistical analysis

To evaluate the outcome events between patients administered DOAC and those receiving LMWH, we determined the risk ratios (RRs) along with their 95 % confidence intervals (95 % CI) for each relevant dichotomous outcome through an inverse variance meta-analysis. For the qualitative assessment of heterogeneity, I^2^ values exceeding 50 % were deemed indicative of substantial heterogeneity [28]. Given the expectation of considerable heterogeneity among the studies, a random effects model was employed to assess and compare the effect sizes of the treatments [29]. A sensitivity analysis was conducted by systematically excluding each study (leave one out) to examine its impact on the overall effect size. Additionally, exploratory subgroup analyses were performed to identify possible sources of heterogeneity for primary outcomes, including VTE and total bleeding, based on cancer type, the specific DOAC administered, follow-up duration, and study design. To evaluate the impact of key clinical and methodological variables on the overall variability detected in the meta-analyses, we also conducted meta-regression analyses. The analyzed predictor variables comprised the publication year, average participant age, sample size, mean body mass index (BMI), male proportion, and the percentages of individuals with advanced-stage cancer and metastatic disease. Publication bias was evaluated for primary outcomes of VTE and total bleeding through the use of funnel plots for visual inspection and the application of Egger's regression test [30]. Statistical analyses were performed using R software version 4.3.3 and the “meta” package [31].

## 3 Results

**Fig. 1:**
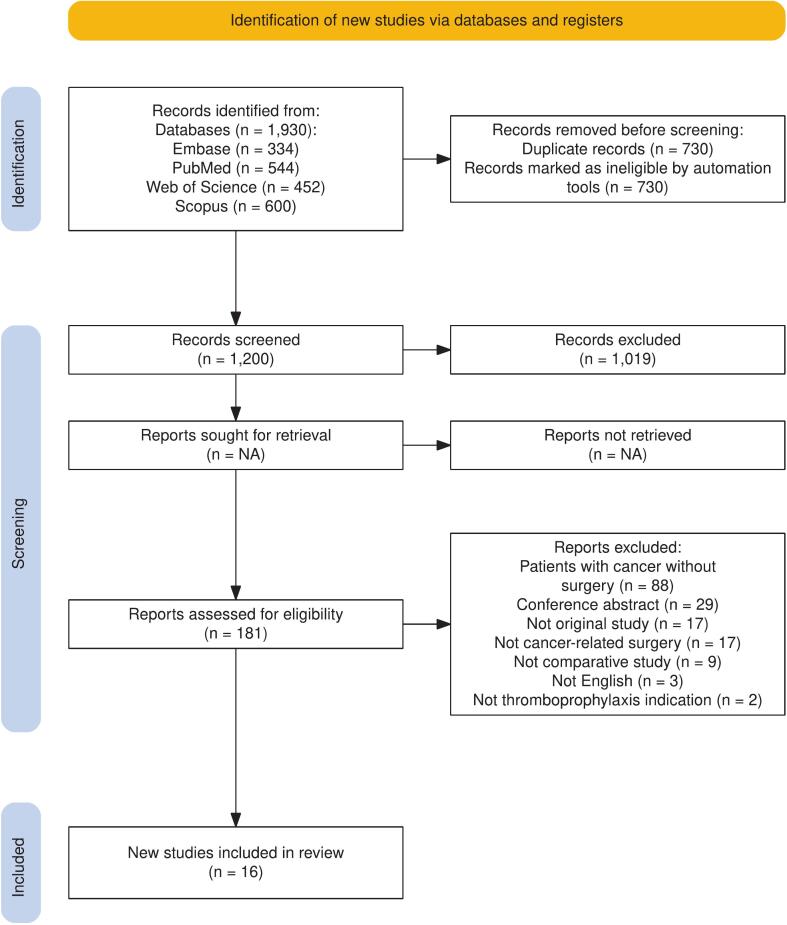
PRISMA chart.

Our systematic database search identified 1930 studies. Following duplicate removal and title/abstract screening, 181 studies were selected for full-text screening. The application of exclusion criteria resulted in 16 studies being included in the final meta-analysis. The study selection process is depicted in the PRISMA chart (Fig. 1).

### 3.1 Baseline characteristics

The final analysis comprised 16 studies, including 3 RCTs [16,32,33] and 13 cohort studies [[34], [35], [36], [37], [38], [39], [40], [41], [42], [43], [44], [45], [46]]. A strength of this meta-analysis is the recency of the included studies, all published within the past six years. Follow-up periods ranged from 1 to 12 months. All studies initiated anticoagulant therapy at discharge or within a maximum of three days post-surgery. The included studies investigated thromboprophylaxis across various cancer types, predominantly gynecological, urological, prostate, bone or soft tissue sarcoma, lung, pancreatic, and lower extremity neoplasms. Surgical approaches included both open surgery and laparoscopy. Detailed data on anticoagulant type, dosage, and duration for both the direct oral anticoagulant (DOAC) and low-molecular-weight heparin (LMWH) groups are presented in Table 1.

**Table 1:** Baseline characteristics of included studies (Abbreviations: BD = twice daily; DOAC = direct oral anticoagulant; LMWH: low-molecular-weight heparin; N: number, RCT = randomized controlled trial; SC: subcutaneous).

Study characteristics						Case group				Control group			
First author/ year of publication	Country	Design	Cancer type	Surgery type	Follow-up	N	Anticoagulant name and dosage	Mean age	Male (%)	N	Anticoagulant name and dosage	Mean age	Male (%)
Chen 2024 [34]	USA	Retrospective cohort studies (database analysis)	Neoplastic pathologic fractures of the lower extremities	Total hip arthroplasty, total knee arthroplasty, and open reduction internal fixation	3 months	706	Apixaban or Rivaroxaban	67.98	41.21	1412	Enoxaparin	68.1	40.22
Diamond 2024 [35]	USA	Retrospective cohort study	Gynecological cancers	Laparotomy	3 months	150	Apixaban for 28 days	59	0	65	Enoxaparin 40 mg daily for 28 days	63	0
Floyd 2024 [36]	USA	Retrospective cohort study	Gynecological cancer	Gynecological cancer surgery	3 months	490	Apixaban 2.5 mg daily for 14 or 28 days	59 ± 12.2	0	138	Enoxaparin 40 mg daily for 28 days	59.5 ± 11.5	0
Knisely 2024 [37]	USA	Retrospective cohort	Gynecological cancers	Laparotomy	3 months	348	Apixaban 2.5 mg BD	56.7 ± 2.1	0	104	Enoxaparin 40 mg	59.9 ± 4	0
Stewart 2024 [38]	Canada	Prospective cohort	Gynecological cancers	Laparotomy	1.5 month	106	Apixaban 2.5 mg BD for 28 days	NA	0	21	Enoxaparin 40 mg for 28 days	NA	0
LiBrizzi 2023 [39]	USA	Retrospective cohort	Bone and soft tissue sarcoma	Surgical resection of primary bone or soft tissue sarcoma	3 months	594	Rivaroxaban, Edoxaban, Apixaban, Betrixaban, Dabigatran, or Argatroban	62.9 ± 12	52.19	1489	Enoxaparin, Dalteparin, or Tinzaparin	53.1 ± 10.2	49.09
Rich 2023 [40]	USA	Retrospective cohort	Urological cancers	Robotic-assisted radical cystectomy with intracorporeal urinary diversion	3 months	124	Apixaban 2.5 mg BD for 21 days	69 ± 2.3	82	250	Enoxaparin 40 mg for 21 days	69.2 ± 2.3	76
Spénard 2023 [41]	Canada	Retrospective cohort	Gynecological cancers	Hysterectomy and cytoreductive procedures	1 month	112	Apixaban 2.5 mg BD for 28 days	59.7 ± 10.8	0	144	Enoxaparin 40 mg for 28 days	60.7 ± 11.2	0
Tasaka 2023 [42]	Japan	Retrospective cohort	Gynecological cancers	Laparotomy	21 days	79	Various DOACs	NA	0	681	Various LMWH	NA	0
Zhao 2023 [32]	China	RCT	Lung cancers	Lobectomy and segmentectomy	1 month	200	Rivaroxaban 10 mg daily	61.7 ± 10.1	53.5	203	Nadroparin 57 units/kg daily until discharge	60.7 ± 9.1	47.3
Johnson 2022 [43]	USA	Retrospective cohort studies (database analysis)	Prostate Cancer	Total Hip Arthroplasty	12 months	2903	Apixaban or Rivaroxaban	NA	NA	768	Enoxaparin or Dalteparin	NA	NA
Oliveira 2022 [33]	Brazil	RCT	Gynecological cancers	Staging surgery, debulking surgery, total or radical hysterectomy, unilateral or bilateral salpingo-oophorectomy, omentectomy, lymph node removal, and open or laparoscopic access	1 month	114	Rivaroxaban 10 mg daily for 30 days	54.5 ± 12.5	0	114	Enoxaparin 40 mg daily for 30 days	53.5 ± 12.1	0
Westerman 2022 [44]	USA	Prospective cohort	Urological cancers	Radical cystectomy, lymph node dissection, radical prostatectomy, open nephrectomies, and partial nephrectomies	1 month	154	Apixaban 2.5 mg BD	66 ± 2.6	84.4	161	Enoxaparin 40 mg	65.2 ± 2.4	79
Ortiz 2021 [45]	USA	Retrospective Feasibility Study	Urological cancer	Robot-assisted radical cystectomy	3 months	29	Rivaroxaban 10 mg or 20 mg daily, Apixaban 5 mg daily or 2.5 mg BD, or dabigatran 75 mg BD	64.5 ± 9.8	69	37	Enoxaparin 30 or 40 mg daily, 60 or 80 mg BD	66.5 ± 8.9	70
Guntupalli 2020 [16]	USA	RCT	Gynecological cancers	Laparotomy and laparoscopy	3 months	204	Apixaban 2.5 mg BD for 28 days	56 ± 12	0	196	Enoxaparin 40 mg daily for 28 days	56 ± 13	0
Rashid 2018 [46]	USA	prospective cohort study	Pancreatic cancer	Pancreaticoduodenectomy, distal Pancreatectomy, irreversible electroporation with Whipple or distal pancreatectomy, and total Pancreatectomy	3 months	87	Dabigatran 220 mg daily for 28 days	NA	NA	18	Enoxaparin for 28 days	NA	NA

Included studies encompassing 12,201 participants, with 6400 receiving DOACs as thromboprophylaxis (case group) and 5801 receiving LMWH (control group). The mean age was 62.05 years in the DOAC group and 60.78 years in the LMWH group. Gender distribution indicated 28.15 % males in the DOAC group and 34.65 % in the LMWH group. This lower proportion of males is attributed to the inclusion of gynecological cancer studies.

### 3.2 Quality assessment

Quality assessment of the observational studies using the NOS indicated that 9 studies were of good quality [34,[36], [37], [38], [39], [40],^43^,^45^,^46^] and 4 were of fair quality [35,41,42,44], primarily due to lower scores in the selection and comparability domains. For the RCTs, the RoB2 tool indicated low concern for bias in two studies [16,32] and some concern in one study [33], specifically within the randomization domain. A comprehensive overview of the quality assessment for all included studies is available in Supplementary Tables 2–3.

### 3.3 Primary outcomes

#### 3.3.1 VTE

**Fig. 2:**
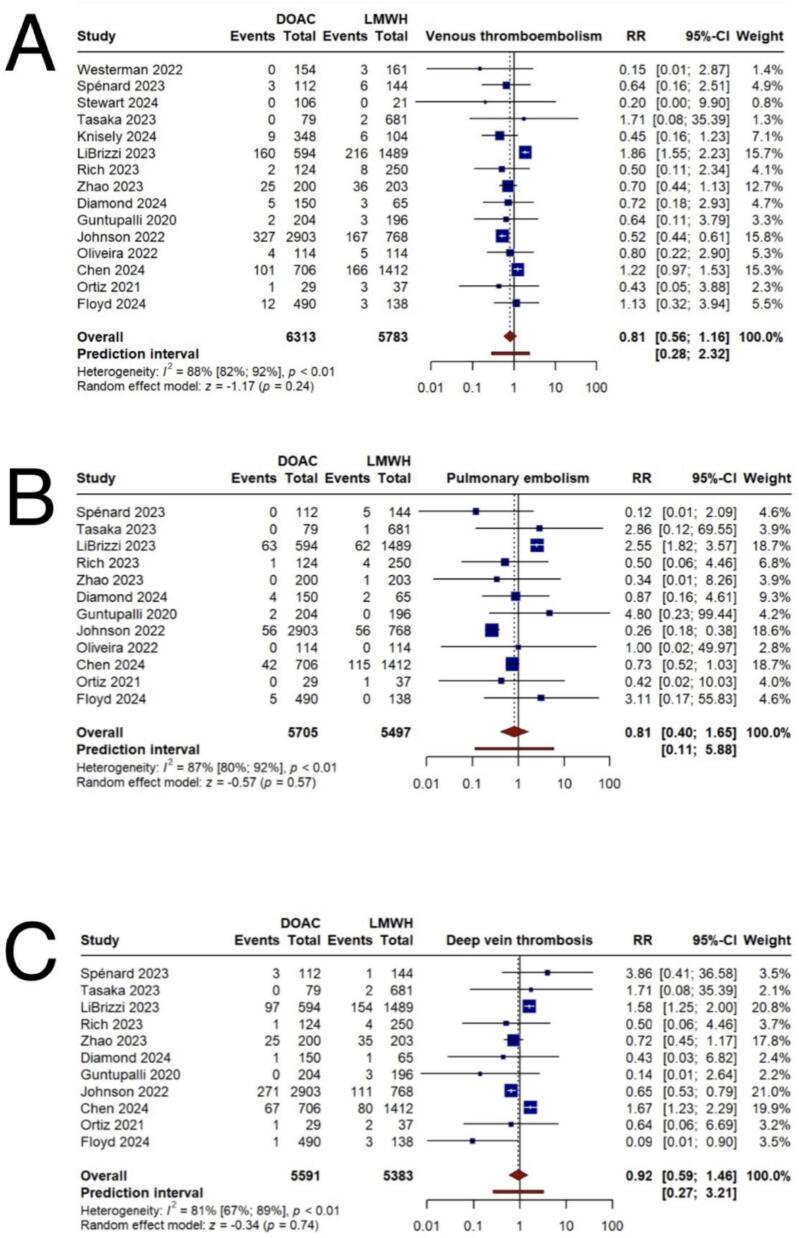
Primary efficacy outcomes. A) incidence of venous thromboembolism B) incidence of pulmonary embolism C) incidence of deep vein thrombosis.

The overall analysis of 15 studies comparing VTE incidence between the DOAC and LMWH groups did not demonstrate a statistically significant difference (RR = 0.81, 95 % CI 0.56;1.16) (Fig. 2A). Additionally, PE and DVT similarly showed no significant differences between the groups, based on 12 studies for PE (RR = 0.81, 95 % CI 0.40;1.65) and 11 studies for DVT (RR = 0.92, 95 % CI 0.59;1.46) (Fig. 2B–C). Given the observed heterogeneity, subgroup analyses were conducted based on cancer type, follow-up duration, specific DOAC administered, and study design. Analysis by cancer type suggested a possible reduction in VTE with DOACs in urological cancer surgeries (RR = 0.52, 95 % CI 0.44;0.61). Subgroup analyses based on follow-up durations of one and three months, by DOAC type (apixaban or other DOACs), and study design (RCT or not-RCT) showed no statistically significant differences in VTE incidence between the treatment groups (Supplementary Figs. 1–4) (Table 2, Table 3).

**Table 2:** Outcomes (Abbreviations: CI = confidence interval; CRNMB = clinically relevant non-major bleeding; DVT = deep vein thrombosis; PE = pulmonary embolism; RR = risk ratio; VTE = venous thromboembolism).

Outcome	Number of studies included	RR (95 % CI)	I^2^	p-value heterogeneity	p-value outcome
**Primary outcomes**					
Total VTE	15	0.81 (0.56;1.16)	88 %	<0.01	0.24
DVT	11	0.92 (0.59;1.46)	81 %	<0.01	0.74
PE	12	0.81 (0.4;1.65)	87 %	<0.01	0.57
Total bleeding	14	0.91 (0.70;1.18)	0 %	0.58	0.49
Major bleeding	12	1.11 (0.67;1.86)	0 %	0.95	0.69
CRNMB	6	0.79 (0.56;1.13)	0 %	0.69	0.20

**Secondary outcomes**					
All-cause mortality	4	1.05 (0.77;1.43)	0 %	0.93	0.76
Hospitalization	7	1.16 (0.98;1.37)	0 %	0.52	0.08

**Table 3:** Subgroup analyses of outcomes based on study design, DOAC type administration, follow-up duration, and cancer type (Abbreviations: CI = confidence interval; DOAC = direct oral anticoagulant; RCT = randomized controlled trial; RR = risk ratio; VTE = venous thromboembolism).

Outcome	Number of studies included	RR (95 % CI)	I^2^	p-value heterogeneity
**VTE**				
*Subgroup based on cancer type:*				
Gynecological cancers	8	0.68 (0.41;1.14)	0 %	0.95
Urological cancers	4	0.52 (0.44;0.61)	0 %	0.87
Others	3	1.21 (0.71;2.05)	89 %	<0.01
*Subgroup based on follow-up duration:*				
One month	6	0.69 (0.46;1.04)	0 %	0.87
Three months	9	0.85 (0.54;1.34)	93 %	<0.01
*Subgroup based on DOAC administered:*				
Apixaban	8	0.59 (0.35;1.01)	0 %	0.92
Other DOACs	7	0.93 (0.58;1.50)	94 %	<0.01
*Subgroup based on study design:*				
RCT	3	0.71 (0.46;1.09)	0 %	0.98
Not-RCT design	12	0.82 (0.53;1.26)	90 %	<0.01

**Total bleeding**				
*Subgroup based on cancer type:*				
Gynecological cancers	7	0.75 (0.53;1.04)	0 %	0.65
Urological cancers	3	0.90 (0.36;2.23)	0 %	0.50
Others	4	1.25 (0.75;2.07)	0 %	0.49
*Subgroup based on follow-up duration:*				
One month	5	1.04 (0.62;1.74)	33 %	0.20
Three months	9	0.80 (0.58;1.10)	0 %	0.88
*Subgroup based on DOAC administered:*				
Apixaban	7	0.64 (0.44;0.94)	0 %	0.86
Other DOACs	7	1.17 (0.85;1.61)	0 %	0.79
*Subgroup based on study design:*				
RCT	3	1.06 (0.60;1.85)	58 %	0.09
Not-RCT design	11	0.81 (0.58;1.12)	0 %	0.86

#### 3.3.2 Total bleeding

**Fig. 3:**
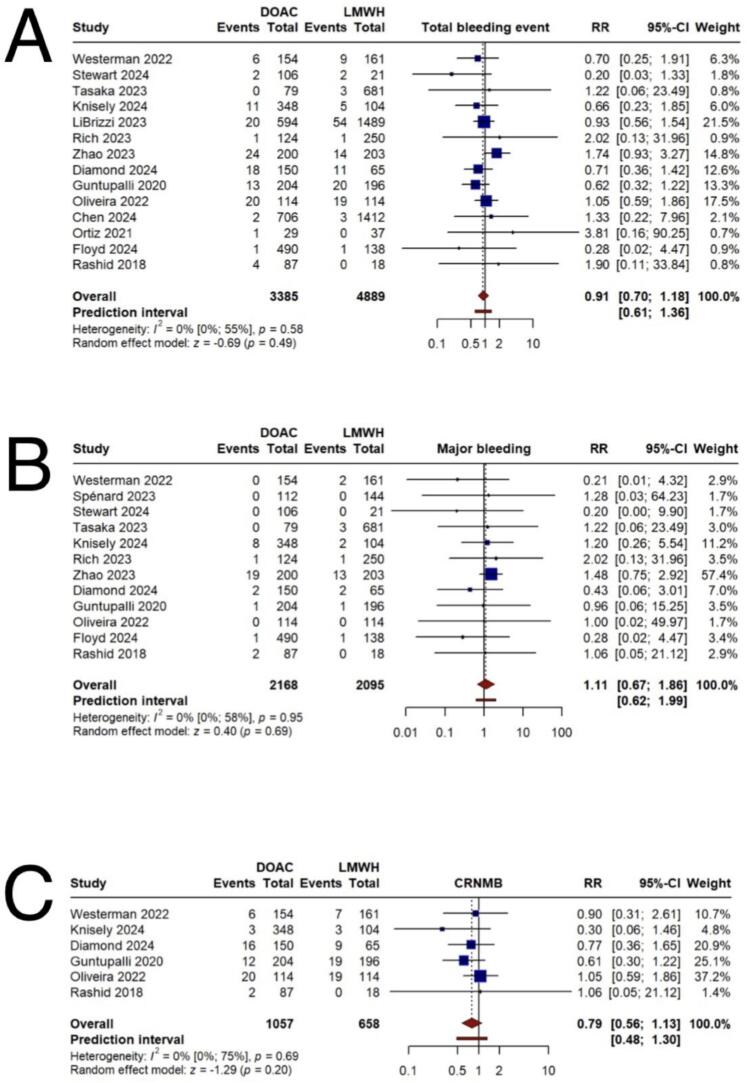
Primary safety outcomes. A) incidence of total bleeding B) incidence of major bleeding C) incidence of clinically relevant non-major bleeding.

The primary safety outcome, total bleeding, showed no significant difference between DOACs and LMWH (RR = 0.91, 95 % CI 0.70;1.18) based on a meta-analysis of 14 studies (Fig. 3A). Stratification of bleeding events into major bleeding and CRNMB resulted in non-significant differences for both categories: major bleeding (RR = 1.11, 95 % CI 0.67; 1.86) and CRNMB (RR = 0.79, 95 % CI 0.56;1.13) (Fig. 3B–C). However, in the subgroup of patients treated with apixaban, the DOAC group demonstrated a trend toward lower incidence of total bleeding compared to the LMWH group (RR = 0.64, 95 % CI 0.44;0.94) based on seven studies. Subgroup analyses of total bleeding by cancer type, follow-up duration, and study design mirrored the overall findings, showing no statistically significant differences between the treatment groups (Supplementary Figs. 5–8) (Table 2, Table 3).

### 3.4 Secondary outcomes

#### 3.4.1 Mortality

**Fig. 4:**
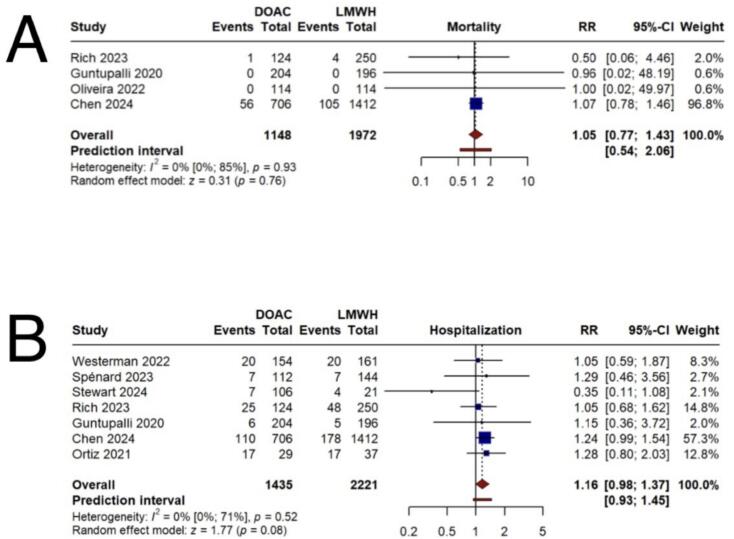
Secondary outcomes. A) incidence of mortality B) incidence of hospitalization.

Comparison of mortality rates between the DOAC and LMWH groups, based on 4 studies, revealed no statistically significant difference (RR = 1.05, 95 % CI 0.77;1.43) (Fig. 4A) (Table 2).

#### 3.4.2 Hospitalization

Analysis of 7 studies evaluating hospitalization rates showed no significant difference between the DOAC and LMWH groups (RR = 1.16, 95 % CI 0.98;1.37) (Fig. 4B) (Table 2).

### 3.5 Sensitivity analysis

To assess the robustness of our findings and minimize the influence of potentially biased studies, LOO sensitivity analyses and removal of outlier studies were performed. After removing the outlier study by LiBrizzi et al. [39], the analysis for VTE demonstrated a statistically significant superior efficacy of DOACs compared to LMWH (RR = 0.71, 95 % CI 0.51;0.97) (Supplementary Figs. 9–10). In the LOO analysis for hospitalization, omitting the study by Stewart et al. [38] resulted in a statistically significant higher hospitalization rate in the DOAC group compared to the LMWH group (RR = 1.19, 95 % CI 1.01;1.41) (Supplementary Fig. 11).

### 3.6 Meta-regression

Meta-regression analysis exploring the influence of baseline characteristics revealed a significant positive association between a higher percentage of patients with metastasis and increased VTE incidence (slope = 0.0088, 95 % CI 0.0013;0.0162, p-value = 0.0211). Conversely, higher percentages of patients with advanced-stage cancer and higher mean BMI were negatively associated with total bleeding incidence (slope = −0.0275, 95 % CI −0.0535;-0.0015, p-value = 0.0383 and slope = −0.1280, 95 % CI −0.2364;-0.0197, p-value = 0.0206, respectively) (Supplementary Tables 4–5).

### 3.7 Publication bias

Funnel plots and Egger's tests for the primary outcomes, VTE and total bleeding, did not indicate significant publication bias among the included studies (VTE Egger test p-value = 0.5551 and total bleeding Egger test p-value = 0.8387, respectively) (Supplementary Figs. 12–13).

## 4 Discussion

This meta-analysis evaluated the efficacy and safety of DOACs versus LMWH for preventing VTE in patients with cancer who underwent cancer-related surgery. Incorporating 16 studies with 12,201 participants, the analysis found no significant difference in VTE incidence between DOAC and LMWH groups. Bleeding rates were also comparable. Notably, subgroup analysis indicated that DOACs significantly reduced VTE incidence in urological cancer patients. Additionally, patients receiving apixaban experienced fewer bleeding events compared to those on LMWH. Mortality and hospitalization rates were similar between the two groups.

The International Society on Thrombosis and Hemostasis (ISTH) guideline recommends extended-duration prophylaxis (4 weeks) with LMWH for high-VTE-risk patients undergoing abdominal or pelvic cancer surgery, provided they are not at high risk for major bleeding [47]. The American Society of Clinical Oncology (ASCO) [20] and the International Thrombosis and Anticoagulation (ITAC) [8] similarly advise at least 7–10 days of anticoagulation, extending to 4 weeks in high-risk cases, with LMWH as the preferred agent. While LMWH remains the standard, recent updates have DOACs as potential alternatives for extended prophylaxis. The 2023 ASCO guidelines included apixaban and rivaroxaban as options for extended thromboprophylaxis, though with a weak recommendation [20]. Similarly, the 2022 ITAC guidelines recommend DOACs such as edoxaban, rivaroxaban, and apixaban for cancer patients with adequate renal function and no high risk of gastrointestinal or genitourinary bleeding [8]. These updates reflect a shift toward DOACs as a more convenient oral alternative to LMWH in select patients.

LMWH primarily functions by inhibiting factor Xa with a minor effect on thrombin inhibition. Its reduced binding to plasma proteins results in a more predictable dose-response relationship, leading to lower rates of heparin-induced thrombocytopenia and osteopenia [48]. However, the need for subcutaneous injections, along with side effects such as injection-site reactions, bruising, bleeding, and nausea, can contribute to suboptimal patient adherence [16,49]. DOACs, in contrast, inhibit either activated thrombin or factor Xa [50] and offer advantages such as oral administration, shorter half-life (allowing flexibility in dosing), and reduced need for routine monitoring, which improves patient adherence and satisfaction [35,50,51]. However, concerns over bleeding risks have limited their widespread adoption.

Our meta-analysis aligns with prior research, demonstrating that DOACs offer a safe and effective alternative to LMWH for postoperative thromboprophylaxis in cancer patients [18,19,52]. The meta-analysis by Zhou et al. (9 studies, 2651 patients) found comparable rates of VTE, major bleeding, and clinically relevant non-major bleeding between DOACs and LMWH for extended thromboprophylaxis [19]. A second meta-analysis by Zhou et al. (10 studies, 3054 patients) further confirmed the comparable efficacy of DOACs and LMWH in preventing VTE in patients following major abdominal or pelvic cancer-related surgery with similar rates of major bleeding and clinically relevant non-major bleeding [18]. A further systematic review and meta-analysis found no significant difference in 30-day VTE incidence or major bleeding rate between DOACs and LMWH in patients undergoing gynecological cancer surgeries [53]. However, DOACs significantly reduced clinically relevant non-major bleeding events [53]. Similarly, in our subgroup analysis patients receiving apixaban experienced a significantly lower incidence of total bleeding compared to those on LMWH.

A comparative effectiveness study on anticoagulant use in cancer patients highlights the growing preference for DOACs over LMWH, despite more than one-third having undergone cancer surgery, though the study did not specifically focus on postoperative settings [54]. Among 5100 patients with cancer-associated thrombosis (CAT), DOACs were prescribed twice as often and were associated with a 50 % lower risk of VTE recurrence, reduced major bleeding, and 60 % lower all-cause mortality compared to LMWH [54]. According to this study, DOACs were less likely to be prescribed in patients with some cancer types including urologic cancers, although, in our study, subgroup analysis revealed a significantly lower VTE incidence in urological cancer patients receiving DOACs.

In our study, we observed that studies with a higher proportion of patients with metastasis had a higher VTE incidence. When adjusting for other risk factors, metastatic stage at diagnosis was found to be a strong independent predictor of developing VTE within the first year after cancer diagnosis [55,56]. Additionally, higher percentages of patients with advanced-stage cancer and elevated mean BMI were associated with a decreased incidence of total bleeding. This may be due to the hypercoagulability associated with obesity [57]. Additionally, anticoagulation targets may be adjusted differently for these patients, potentially leading to lower bleeding rates, though this hypothesis requires further investigation.

This meta-analysis offers several key strengths that enhance the reliability and applicability of its findings. First, the large sample size of 12,201 participants across 16 studies provides robust statistical power. The broad scope of the included studies also allows for a comprehensive assessment of both the efficacy and safety of DOACs compared to LMWH in a wide range of cancer types and surgical settings. Another strength is the detailed subgroup analysis based on follow-up time, type of DOAC, and cancer type. This allowed for a more nuanced evaluation of how different cancer types respond to DOACs and provided valuable insights into potential treatment benefits for specific groups of patients. The main distinctions between our study and previous systematic reviews and meta-analyses are the broader scope, a greater number of included studies and patients, and stricter inclusion criteria. By performing detailed subgroup analyses, our meta-analysis provides a more thorough evaluation of both efficacy and safety outcomes. Furthermore, our study accounts for differences in follow-up durations and DOAC types, improving the relevance of our findings for clinical practice. With the inclusion of more recent and comprehensive data, our analysis offers a robust comparison of DOACs versus LMWH in patients undergoing cancer-related surgeries. Despite its strengths, this meta-analysis has several limitations. One major issue is the heterogeneity among the included studies, which varied in design, patient populations, and treatment protocols. Furthermore, some subgroup analyses had limited sample sizes, which could impact the reliability of findings for these specific populations. Another limitation is the lack of long-term data on the use of DOACs in cancer patients, as the studies predominantly focused on short-term postoperative outcomes.

Future research should include larger, well-powered RCTs that focus on specific cancer subtypes to confirm the findings of this study. Additionally, long-term follow-up studies are needed to evaluate the extended safety and efficacy of DOACs in cancer patients, particularly with regard to VTE recurrence, bleeding complications, and survival and also assess the efficacy of switching from LMWH to DOAC and its optimal time. Furthermore, an important unmet need in this setting is the creation of reliable, tailored risk prediction models such as some developed clinical scores and machine learning algorithms [[58], [59], [60]], to help improve the personalized anticoagulant selection and risk stratification in this vulnerable cancer population.

## 5 Conclusion

This meta-analysis demonstrated no significant difference in overall VTE incidence or bleeding rates between DOACs compared to LMWH for preventing VTE in cancer patients undergoing cancer-related surgery. Subgroup analysis suggested that DOACs significantly reduced VTE incidence in urological cancer patients and apixaban was associated with fewer bleeding events compared to LMWH. However, subgroup findings arise from a relatively small number of studies, and thus should be interpreted with caution as they may be prone to type I error related to multiple comparisons. These observations are best regarded as hypothesis-generating and warrant further investigation in larger, confirmatory trials. Additionally, while current guidelines still favor LMWH, these findings support the growing role of DOACs as a convenient and potentially preferred option in certain cancer subgroups, emphasizing the need for further research to confirm long-term outcomes and refine treatment recommendations.

## Ethical

• We confirm that the work described has not been published previously except in the form of a preprint, an abstract, a published lecture, academic thesis or registered report.
• We confirm that the article is not under consideration for publication elsewhere.
• We confirm that the article's publication is approved by all authors and tacitly or explicitly by the responsible authorities where the work was carried out.
• We confirm that, if accepted, the article will not be published elsewhere in the same form, in English or in any other language, including electronically, without the written consent of the copyright-holder.

## CRediT authorship contribution statement

**Asma Mousavi:** Writing – original draft, Project administration, Methodology, Investigation, Conceptualization. **Shayan Shojaei:** Writing – original draft, Project administration, Methodology, Investigation, Conceptualization. **Parham Dastjerdi:** Writing – original draft. **Soheil Rahmati:** Visualization, Formal analysis. **Kasra Izadpanahi:** Writing – original draft. **Homayoun Pishraft-sabet:** Writing – original draft. **Elmira Jafari Afshar:** Writing – original draft, Supervision. **Keyvan Salehi:** Validation, Investigation. **Mahshad Sabri:** Writing – review & editing. **Mahsa Noohi Arbatan:** Methodology, Investigation. **Parisa Fallahtafti:** Writing – original draft. **Aronow Wilbert:** Writing – review & editing. **Andrew P. Ambrosy:** Writing – review & editing. **Mushabbar A. Syed:** Writing – review & editing. **Mina Iskander:** Writing – review & editing. **Kaveh Hosseini:** Validation, Supervision, Project administration.

## Funding

This research did not receive any specific grant from funding agencies in the public, commercial, or not-for-profit sectors.

## Declaration of competing interest

The authors declare that they have no known competing financial interests or personal relationships that could have appeared to influence the work reported in this paper.

The author is an Editorial Board Member/Editor-in-Chief/Associate Editor/Guest Editor for this journal and was not involved in the editorial review or the decision to publish this article.

## Appendix A Supplementary data

Supplementary material Image 1
