# Supplementary material for: Direct oral anticoagulants versus low-molecular-weight heparin for thromboprophylaxis in cancer-related surgeries: A meta-analysis of efficacy and safety outcomes
Source: Am Heart J Plus. 2025 Sep 11;59:100607. doi: 10.1016/j.ahjo.2025.100607 (PMC12624218; doi:10.1016/j.ahjo.2025.100607)
Supplement: Supplementary file 1 — Supplementary material [file mmc1.docx]

Supplementary materials

| **Database** | **Search query** | **Result** |
| --- | --- | --- |
| **PubMed** | ((((((((((((heparin, low molecular weight[Title/Abstract]) OR (low molecular weight heparin[Title/Abstract])) OR (low-molecular-weight heparin[Title/Abstract])) OR (LMWH[Title/Abstract])) OR (tedelparin[Title/Abstract])) OR (dalteparin[Title/Abstract])) OR (enoxaparine[Title/Abstract])) OR (fraxiparin[Title/Abstract])) OR (nadroparin[Title/Abstract])) OR (tinzaparin[Title/Abstract])) OR (dalteparin[Title/Abstract])) AND (((((((((((((((((apixaban[Title/Abstract]) OR (betrixaban[Title/Abstract])) OR (edoxaban[Title/Abstract])) OR (rivaroxaban[Title/Abstract])) OR (dabigatran[Title/Abstract])) OR (ximelagatran[Title/Abstract])) OR (factor xa inhibitor[Title/Abstract])) OR (non-vitamin k antagonist[Title/Abstract])) OR (non-vitamin k antagonist oral anticoagulant[Title/Abstract])) OR (direct oral anticoagulant[Title/Abstract])) OR (doacs[Title/Abstract])) OR (doac[Title/Abstract])) OR (novel oral anticoagulant[Title/Abstract])) OR (edoxaban[Title/Abstract])) OR (betrixaban[Title/Abstract])) OR (direct-acting oral anticoagulant[Title/Abstract])) OR (direct acting oral anticoagulant[Title/Abstract]))) AND (((((((((((cancer[Title/Abstract]) OR (neoplasm[Title/Abstract])) OR (neoplasia[Title/Abstract])) OR (neoplasias[Title/Abstract])) OR (cancers[Title/Abstract])) OR (malignant[Title/Abstract])) OR (malignancy[Title/Abstract])) OR (malignancies[Title/Abstract])) OR (tumor[Title/Abstract])) OR (tumour[Title/Abstract])) OR (tumors[Title/Abstract])) | 544 |
| **Scopus** | ( TITLE-ABS-KEY ( tumor ) OR TITLE-ABS-KEY ( neoplasm ) OR TITLE-ABS-KEY ( carcinoma ) OR TITLE-ABS-KEY ( cancer ) OR TITLE-ABS-KEY ( malignant ) OR TITLE-ABS-KEY ( tumour ) OR TITLE-ABS-KEY ( neoplasia ) OR TITLE-ABS-KEY ( malignancy ) OR TITLE-ABS-KEY ( malignancies ) AND TITLE-ABS-KEY ( apixaban ) OR TITLE-ABS-KEY ( betrixaban ) OR TITLE-ABS-KEY ( edoxaban ) OR TITLE-ABS-KEY ( rivaroxaban ) OR TITLE-ABS-KEY ( dabigatran ) OR TITLE-ABS-KEY ( ximelagatran ) OR TITLE-ABS-KEY ( "factor xa inhibitor" ) OR TITLE-ABS-KEY ( "non-vitamin k antagonist oral anticoagulant" ) OR TITLE-ABS-KEY ( "non-vitamin k antagonist" ) OR TITLE-ABS-KEY ( "direct oral anticoagulant" ) OR TITLE-ABS-KEY ( doacs ) OR TITLE-ABS-KEY ( doac ) OR TITLE-ABS-KEY ( "novel oral anticoagulant" ) OR TITLE-ABS-KEY ( edoxaban ) OR TITLE-ABS-KEY ( betrixaban ) OR TITLE-ABS-KEY ( "direct-acting oral anticoagulant" ) OR TITLE-ABS-KEY ( "direct acting oral anticoagulant" ) OR TITLE-ABS-KEY ( "direct acting oral anticoagulants" ) AND TITLE-ABS-KEY ( "heparin, low molecular weight" ) OR TITLE-ABS-KEY ( "low molecular weight heparin" ) OR TITLE-ABS-KEY ( "low-molecular-weight heparin" ) OR TITLE-ABS-KEY ( lmwh ) OR TITLE-ABS-KEY ( tedelparin ) OR TITLE-ABS-KEY ( "dalteparin sodium" ) OR TITLE-ABS-KEY ( enoxaparine ) OR TITLE-ABS-KEY ( fraxiparin ) OR TITLE-ABS-KEY ( nadroparin ) OR TITLE-ABS-KEY ( tinzaparin ) OR TITLE-ABS-KEY ( dalteparin ) ) | 600 |
| **Embase** | ('tumor':ti,ab,kw OR 'tumors':ti,ab,kw OR 'neoplasm':ti,ab,kw OR 'carcinoma':ti,ab,kw OR 'malignancy':ti,ab,kw OR 'malignant neoplasm':ti,ab,kw) AND ('apixaban':ti,ab,kw OR 'betrixaban':ti,ab,kw OR 'edoxaban':ti,ab,kw OR 'rivaroxaban':ti,ab,kw OR 'dabigatran':ti,ab,kw OR 'ximelagatran':ti,ab,kw OR 'blood clotting factor 10a inhibitor':ti,ab,kw OR 'non vitamin k antagonist oral anticoagulant':ti,ab,kw OR 'non vitamin k oral anticoagulant':ti,ab,kw OR 'direct acting oral anticoagulant':ti,ab,kw) AND ('low molecular weight heparin':ti,ab,kw OR 'enoxaparin':ti,ab,kw OR 'nadroparin':ti,ab,kw OR 'dalteparin':ti,ab,kw OR 'tinzaparin':ti,ab,kw) | 334 |
| **Web of Science** | ((AB=(tumor OR neoplasm OR cancer OR carcinoma OR malignant OR tumour OR tumor OR neoplasia OR malignancy OR malignancies)) AND AB=(apixaban OR betrixaban OR edoxaban OR rivaroxaban OR dabigatran OR ximelagatran OR "factor xa inhibitor" OR "non-vitamin k antagonist oral anticoagulant" OR "non-vitamin k antagonist" OR "direct oral anticoagulant" OR doac OR doacs OR "novel oral anticoagulant" OR edoxaban OR betrixaban OR "direct-acting oral anticoagulant" OR "direct acting oral anticoagulant")) AND AB=("heparin, low molecular weight" OR "low molecular weight heparin" OR "low-molecular-weight heparin" OR LMWH OR tedelparin OR dalteparin OR enoxaparine OR fraxiparin OR nadroparin OR tinzaparin OR dalteparin) | 452 |
| **Total** |  | 1930 |

Supplementary Table 1. Search terms of each database

| **Study** | **Domain 1** | **Domain 2** | **Domain 3** | **Domain 4** | **Domain 5** | **Overall** |
| --- | --- | --- | --- | --- | --- | --- |
| Zhao  2023 |  |  |  |  |  |  |
| Oliveira 2022 |  |  |  |  |  |  |
| Guntupalli 2020 |  |  |  |  |  |  |

Supplementary Table 2. Quality assessment of randomized controlled trials with Risk of Bias 2 tool.

|  | **Selection** | | | | **Comparability** | **Outcome** | | | **Overall risk of bias** |
| --- | --- | --- | --- | --- | --- | --- | --- | --- | --- |
| First author/  publication year | Representativeness of the exposed cohort | Selection of the non-exposed cohort | Ascertainment of exposure | Demonstration that outcome of interest was not present at start of study |  | Assessment of outcome | Was follow-up long enough for outcomes to occur | Adequacy of follow up of cohorts |  |
| Chen 2024 | **** | **** | **** |  | **** | **** | **** | **** | **Good** |
| Diamond 2024 | **** | **** |  |  | **** |  | **** | **** | **Fair** |
| Floyd 2024 | **** | **** | **** |  | **** | **** | **** | **** | **Good** |
| Knisely 2024 | **** | **** | **** |  | **** |  | **** | **** | **Good** |
| Librizzi 2023 | **** | **** | **** |  | **** | **** | **** | **** | **Good** |
| Rich 2023 | **** | **** | **** |  | **** | **** | **** | **** | **Good** |
| Spenard 2023 | **** | **** | **** |  | **** | **** |  | **** | **Fair** |
| Stewart 2023 | **** | **** | **** |  | **** | **** | **** | **** | **Good** |
| Tasaka 2023 | **** | **** | **** |  | **** | **** |  | **** | **Fair** |
| Johnson 2022 |  | **** | **** | **** | **** | **** | **** | **** | **Good** |
| Westerman 2022 | **** | **** | **** |  | **** | **** |  | **** | **Fair** |
| Ortiz 2021 | **** | **** | **** |  | **** | **** | **** | **** | **Good** |
| Rashid 2018 | **** | **** | **** |  | **** | **** | **** | **** | **Good** |

Supplementary Table 3. Quality assessment of cohort studies with Newcastle Ottawa scale.

| **Moderator** | **Slope** | **95% CI** | **p** | **R² (Proportion of Variance Explained)** |
| --- | --- | --- | --- | --- |
| Publication Year | 0.1828 | -0.1791 to 0.5448 | 0.3221 | 8.66% |
| Sample Size | 0.0001 | -0.0002 to 0.0004 | 0.5955 | 0.00% |
| Mean Age | -0.0193 | -0.1005 to 0.0620 | 0.6424 | 0.00% |
| Male (%) | 0.0034 | -0.0109 to 0.0176 | 0.6430 | 3.06% |
| Mean BMI | 0.0003 | -0.1012 to 0.1017 | 0.9960 | 0.00% |
| Metastasis (%) | 0.0088 | 0.0013 to 0.0162 | 0.0211 | 91.78% |
| Advanced Stage (%) | -0.0082 | -0.0306 to 0.0142 | 0.4734 | 0.00% |

Supplementary Table 4. Meta-regression analyses of venous thromboembolism outcome

| **Moderator** | **Slope** | **95% CI** | **p** | **R² (Proportion of Variance Explained)** |
| --- | --- | --- | --- | --- |
| Publication Year | -0.0008 | -0.2129 to 0.2114 | 0.9943 | 0.00% |
| Sample Size | 0.0001 | -0.0003 to 0.0005 | 0.7928 | 0.00% |
| Mean Age | 0.0241 | -0.0482 to 0.0965 | 0.5133 | 0.00% |
| Male (%) | 0.0066 | -0.0025 to 0.0157 | 0.1558 | 27.95% |
| Mean BMI | -0.1280 | -0.2364 to -0.0197 | 0.0206 | 100% |
| Metastasis (%) | -0.0063 | -0.0335 to 0.0208 | 0.6472 | 0.00% |
| Advanced Stage (%) | -0.0275 | -0.0535 to -0.0015 | 0.0383 | 100% |

Supplementary Table 5. Meta-regression analyses of total bleeding outcome


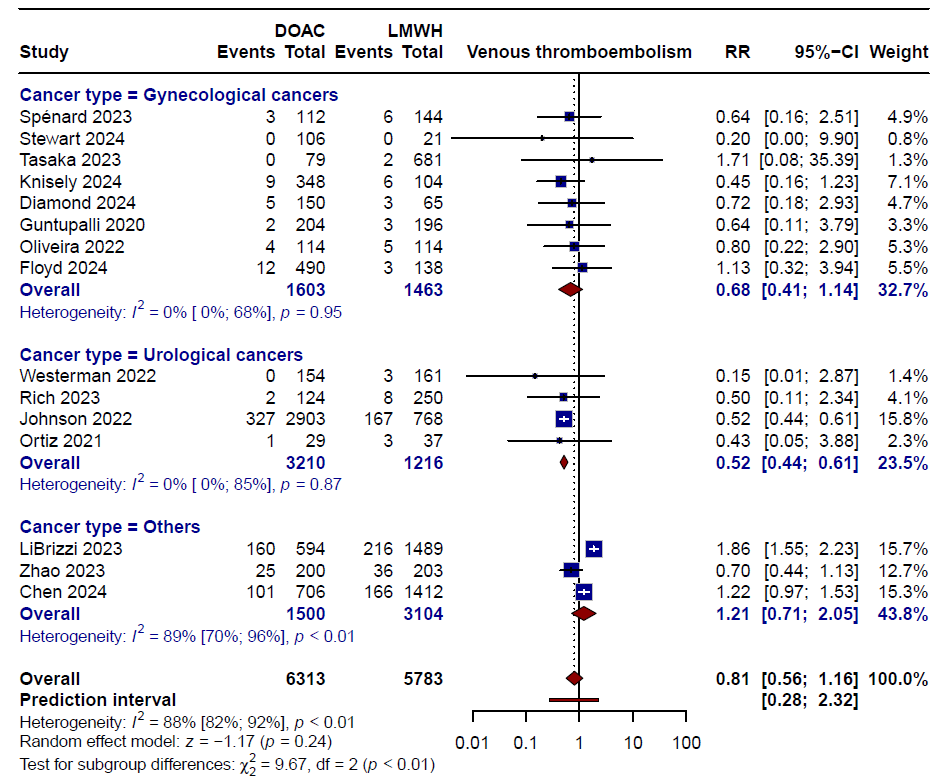


Supplementary Figure 1. Subgroup analysis of venous thromboembolism based on cancer type


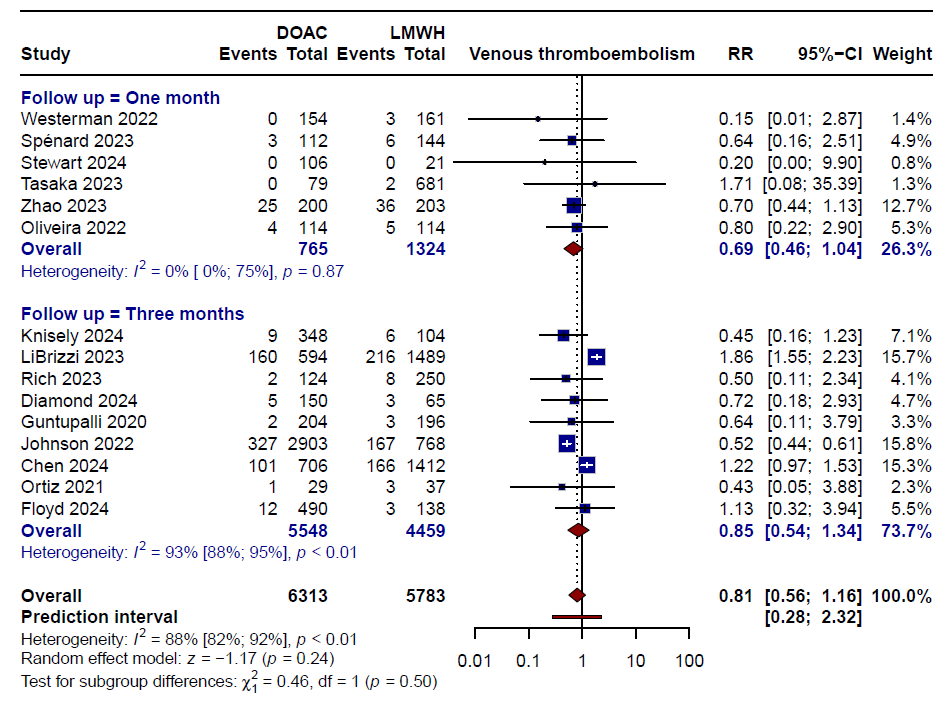


Supplementary Figure 2. Subgroup analysis of venous thromboembolism based on follow-up duration


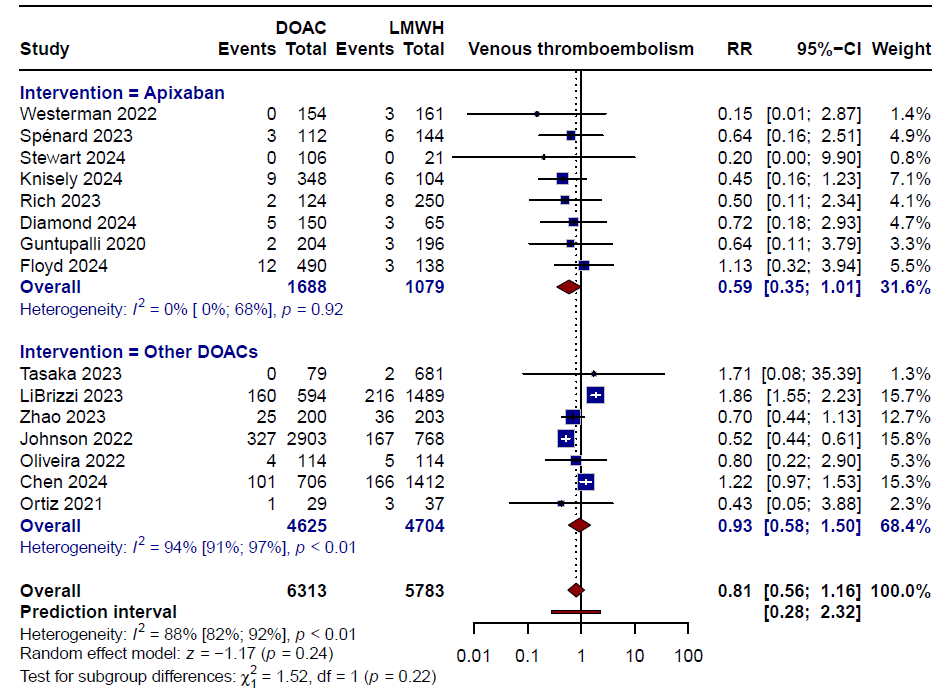


Supplementary Figure 3. Subgroup analysis of venous thromboembolism based on DOAC type


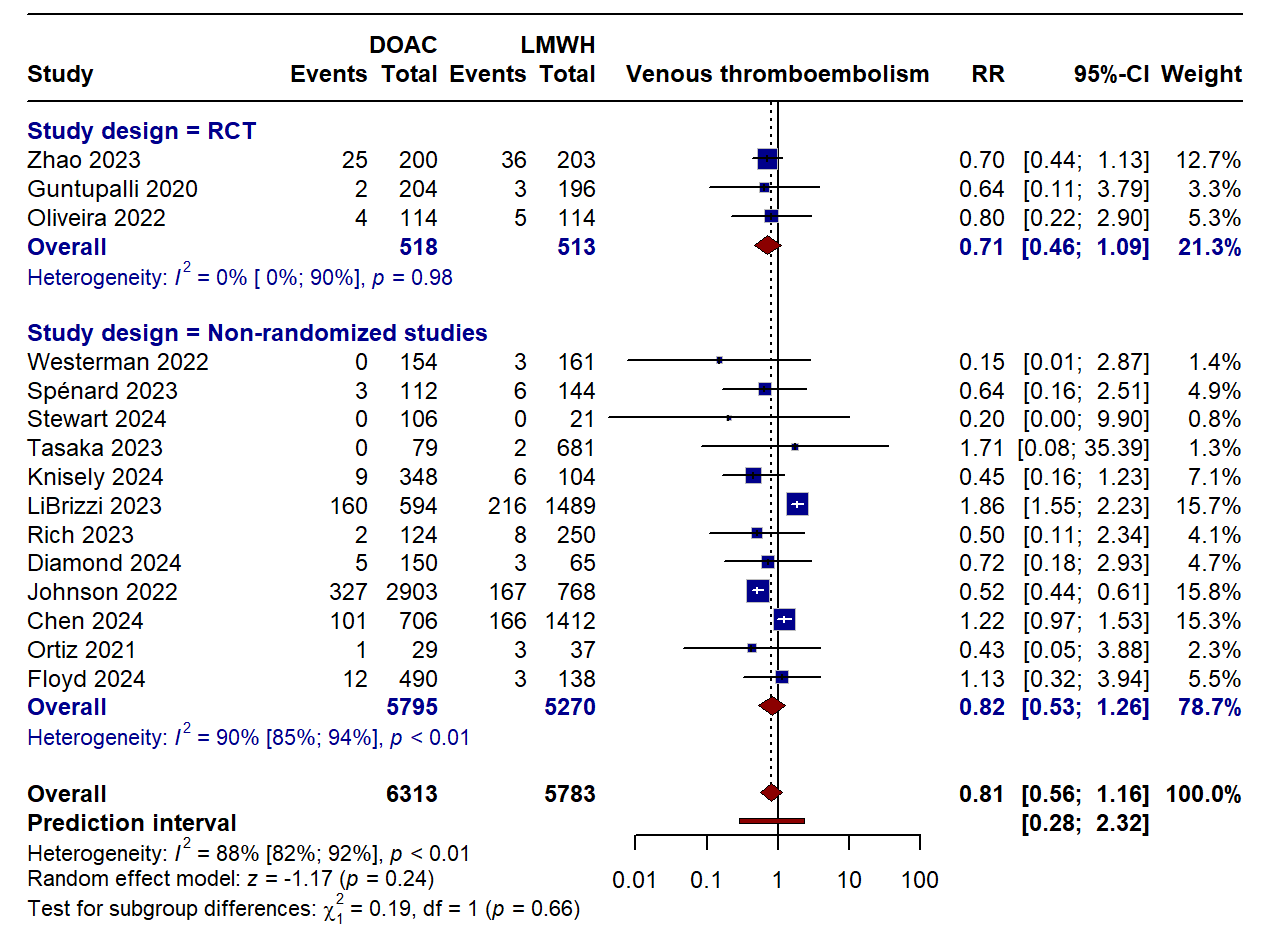


Supplementary Figure 4. Subgroup analysis of venous thromboembolism based on study design


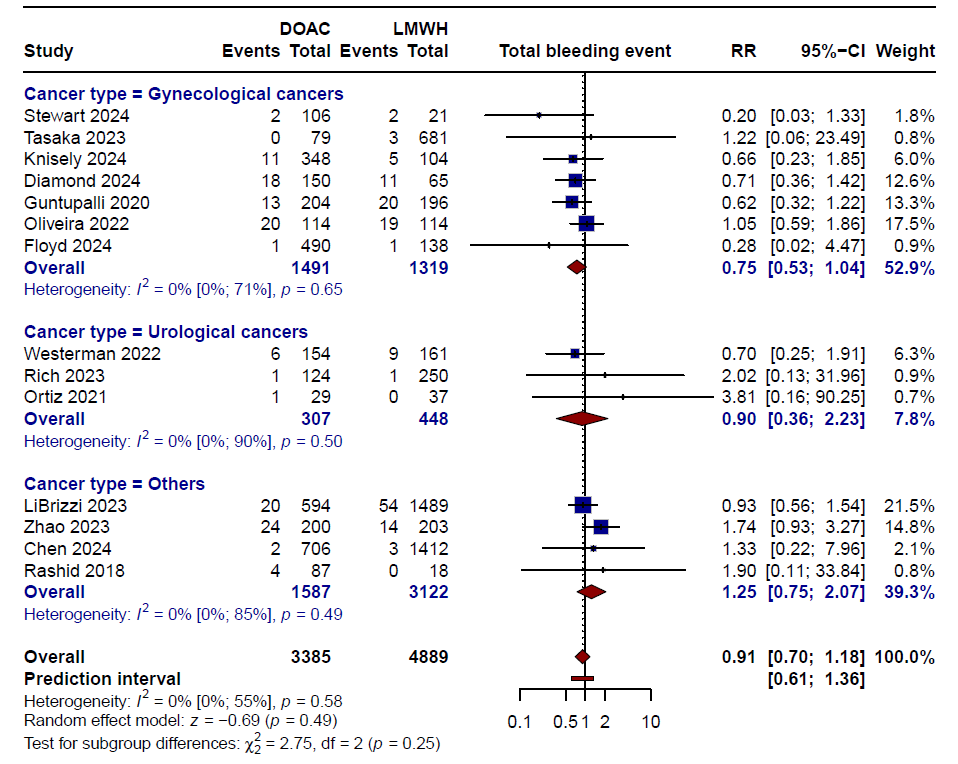


Supplementary Figure 5. Subgroup analysis of total bleeding based on cancer type


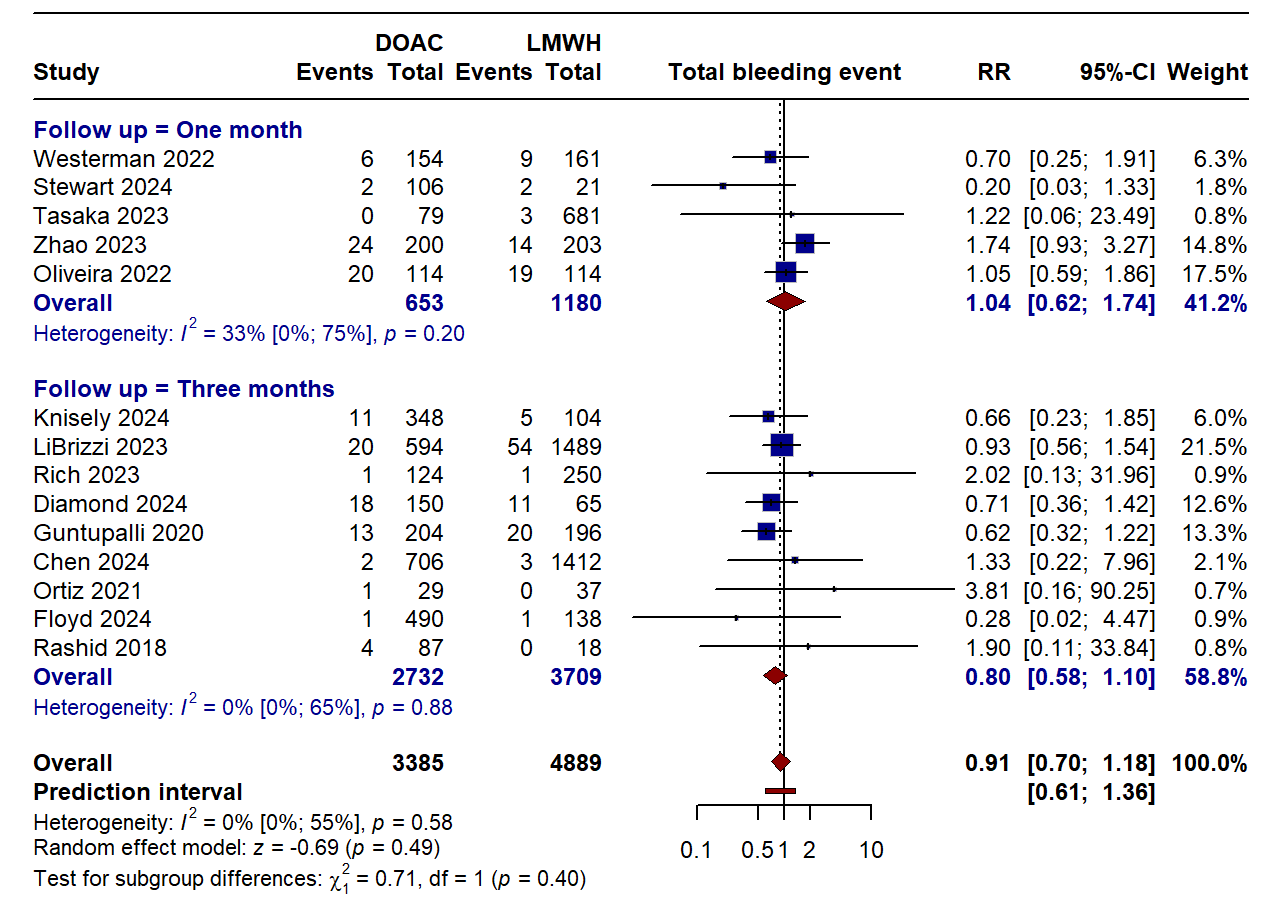


Supplementary Figure 6. Subgroup analysis of total bleeding based on follow-up duration


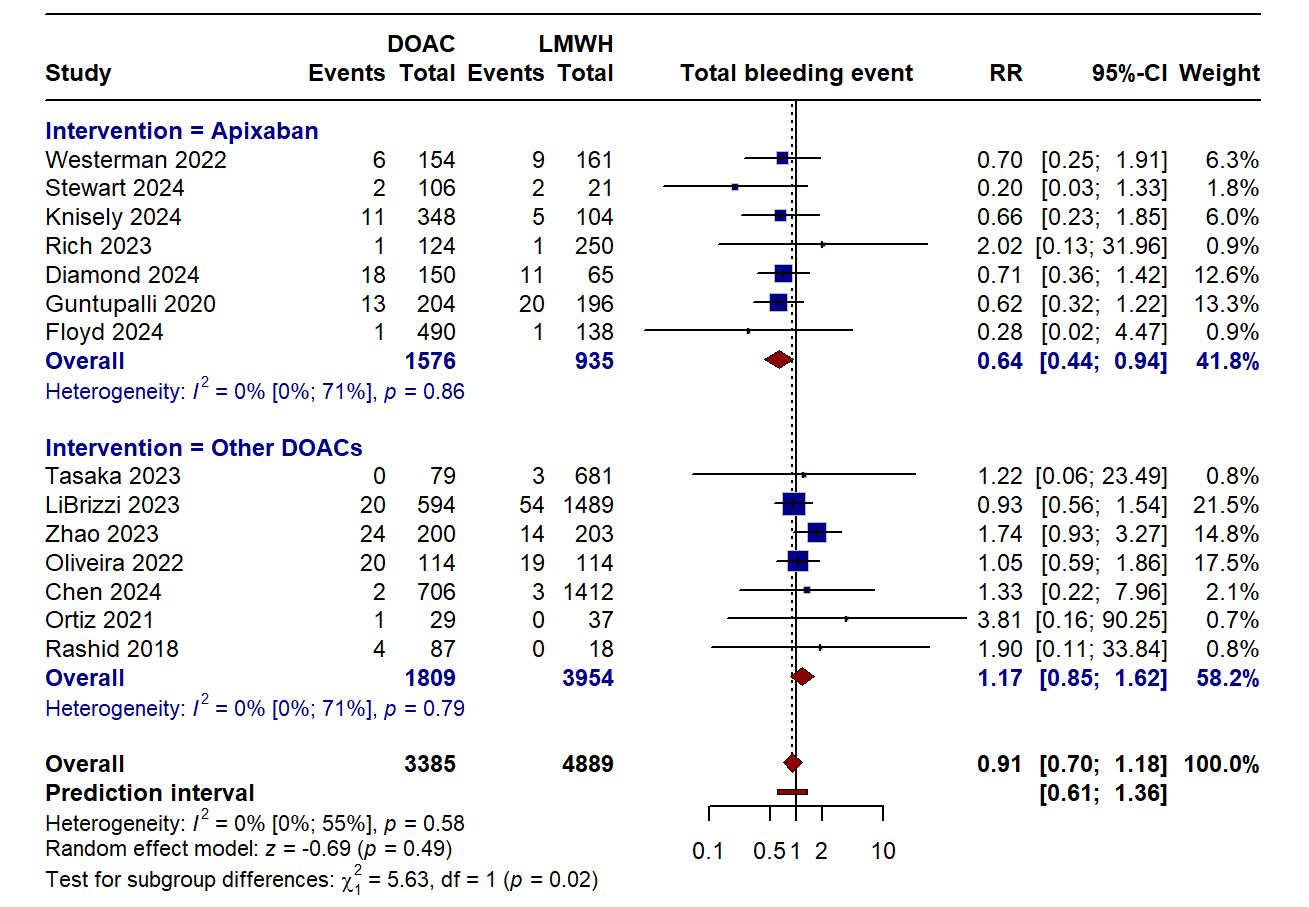


Supplementary Figure 7. Subgroup analysis of total bleeding based on DOAC type


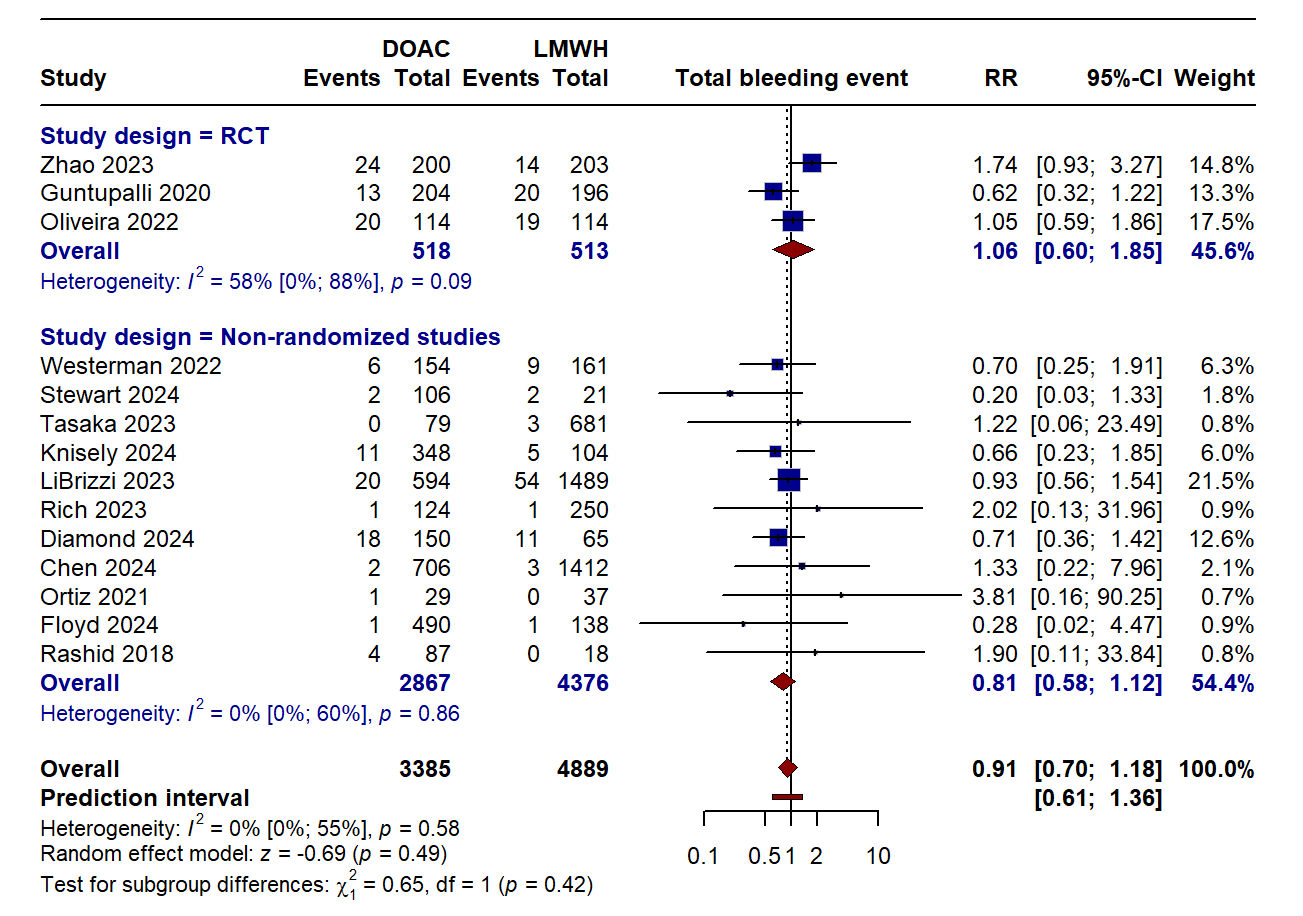


Supplementary Figure 8. Subgroup analysis of total bleeding based on study design


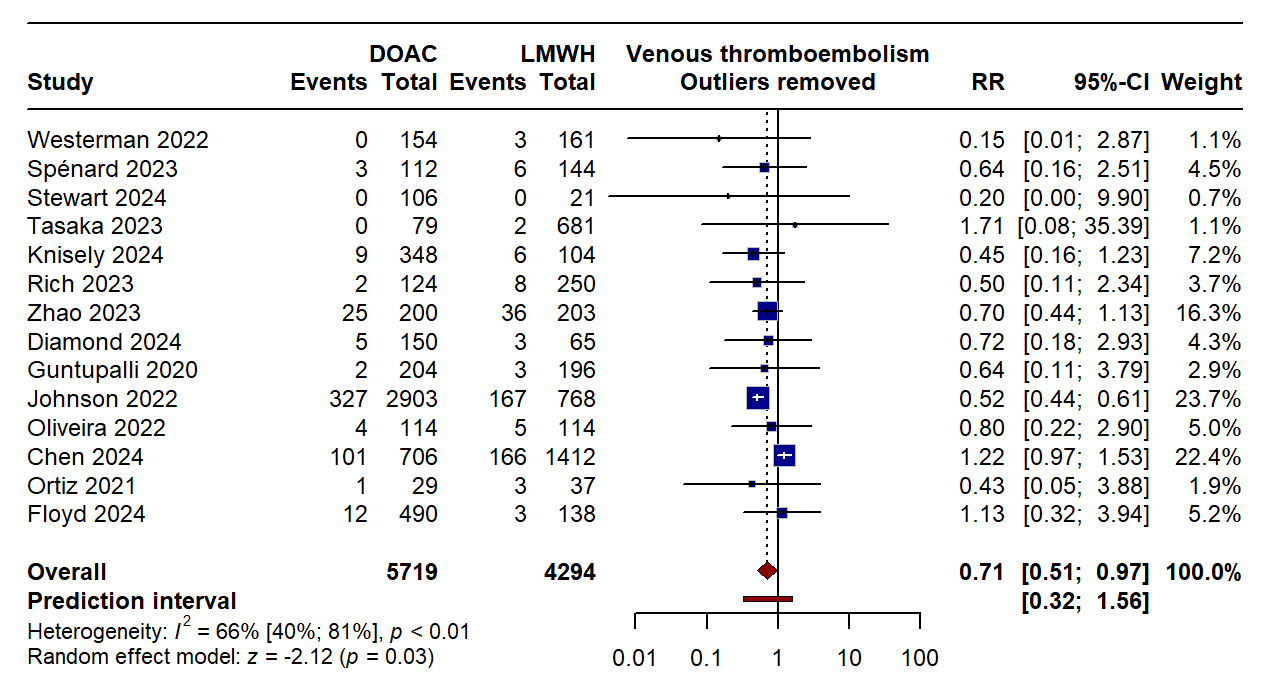


Supplementary Figure 9. Venous thromboembolism incidence meta-analysis by removing the outlier study


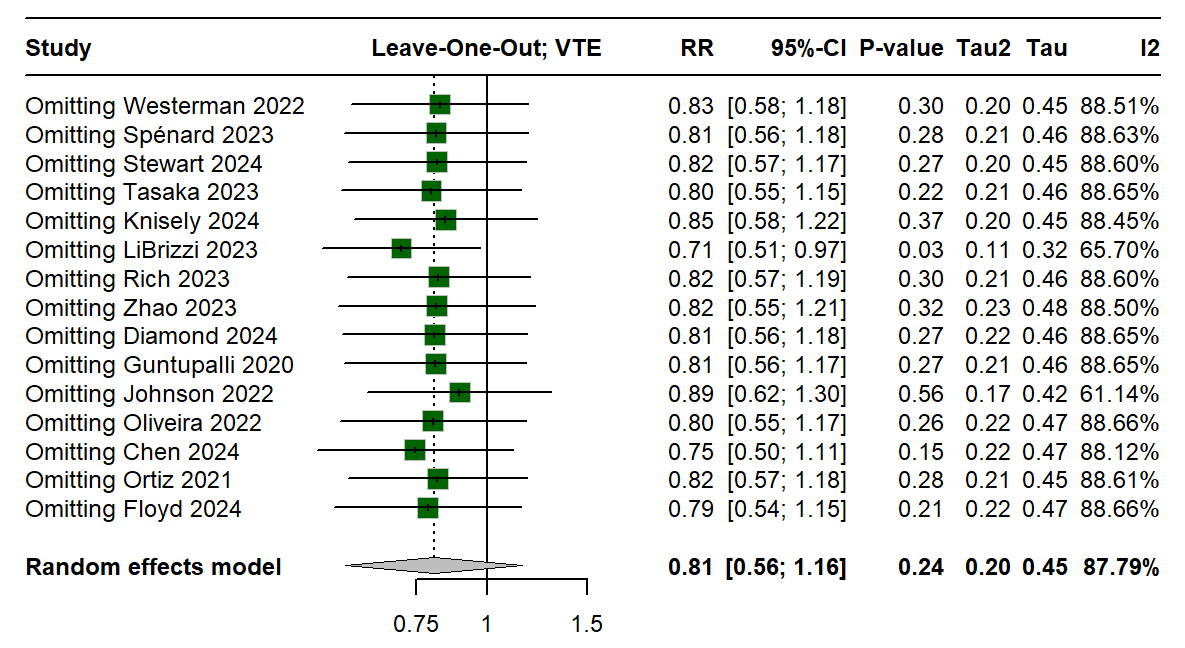


Supplementary Figure 10. Leave-one out sensitivity analysis of venous thromboembolism incidence outcome


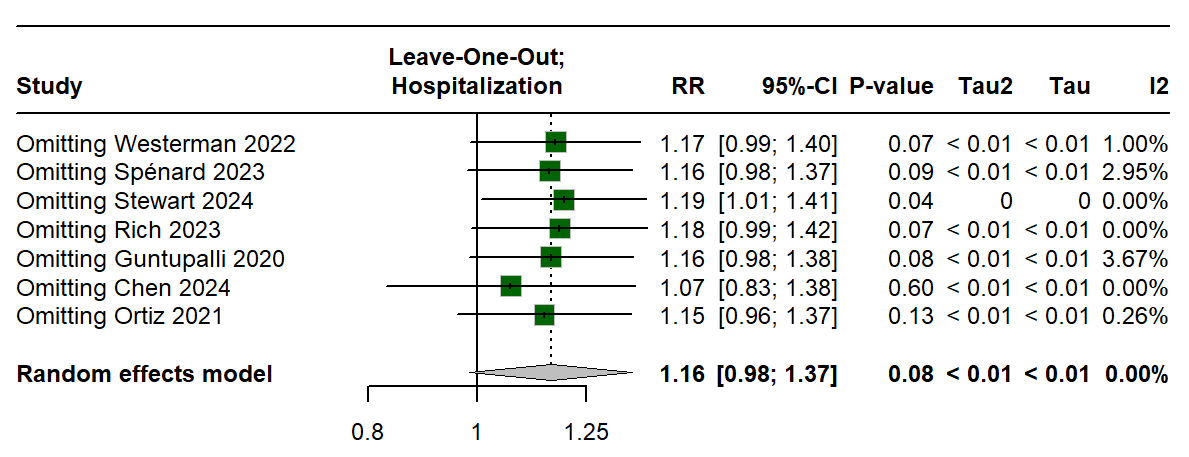


Supplementary Figure 11. Leave-one out sensitivity analysis of hospitalization incidence outcome


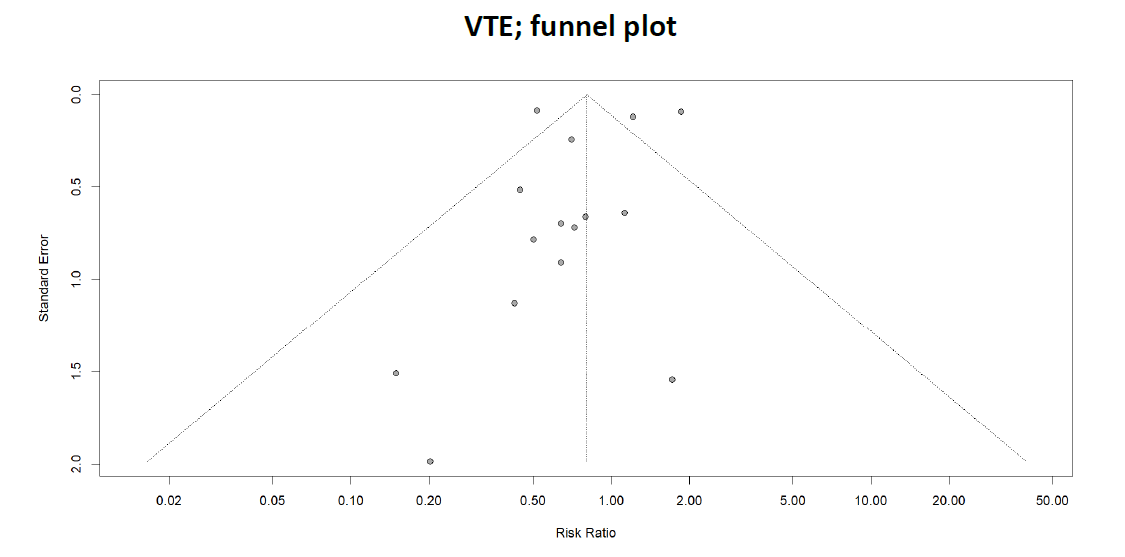


Supplementary Figure 12. Funnel plot for sensitivity analysis of venous thromboembolism meta-analysis


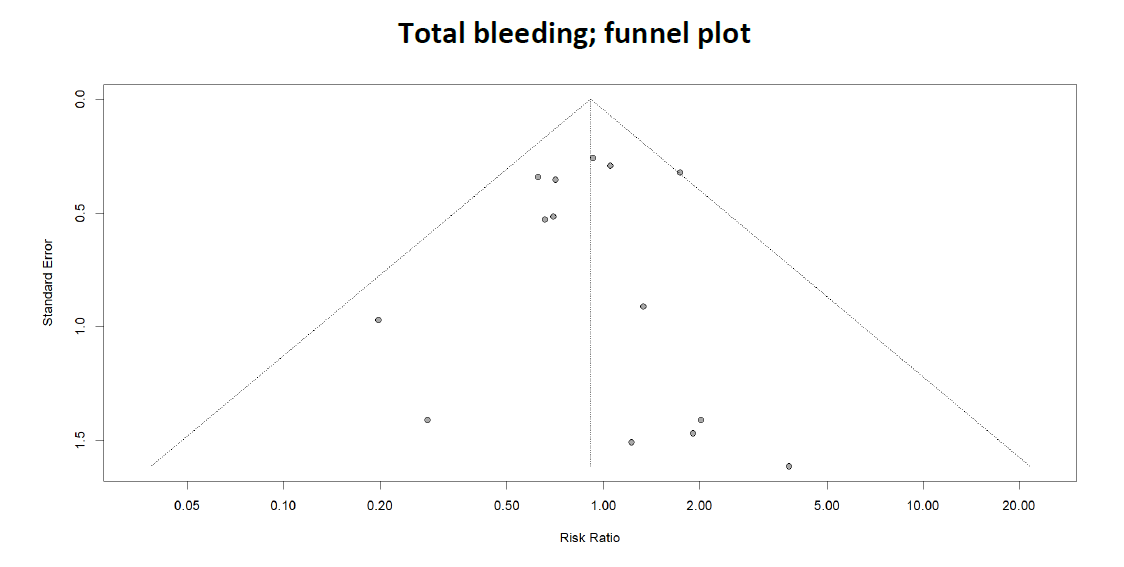


Supplementary Figure 13. Funnel plot for sensitivity analysis of total bleeding meta-analysis
